# The Prevalence of Pancreas Divisum and Its Association with Pancreatic Diseases: A Systematic Review and Meta-Analysis

**DOI:** 10.3390/medicina62050953

**Published:** 2026-05-13

**Authors:** Worawit Suphamungmee, Napawan Taradolpisut, Laphatrada Yurasakpong, Thanyaporn Senarai, Athikhun Suwannakhan

**Affiliations:** 1 Department of Anatomy, Faculty of Science, Mahidol University, Bangkok 10400, Thailand; worawit.sup@mahidol.ac.th (W.S.); napawan.taa@student.mahidol.edu (N.T.); laphatrada.yur@mahidol.ac.th (L.Y.); 2 Department of Anatomy, Faculty of Medicine, Khon Kaen University, Khon Kaen 40000, Thailand; thansen@kku.ac.th; 3 Human Anatomy Unit, Department of Biomedical Sciences, College of Medicine and Health, University of Birmingham, Birmingham B15 2TT, UK

**Keywords:** pancreas, pancreatitis, cholangiopancreatography, meta-analysis

## Abstract

*Background and Objectives*: Pancreas divisum (PD) is the most common congenital anomaly of the pancreatic ductal system and has been suggested to contribute to pancreatic pathology. However, its true prevalence and relationship with pancreatic diseases remain debated. This systematic review and meta-analysis aimed to estimate the global prevalence of PD and evaluate its association with pancreatic disease. *Materials and Methods*: A comprehensive search of Google Scholar, Scopus, and PubMed was conducted to identify studies reporting the prevalence of PD across all populations and diagnostic modalities. Pooled prevalence estimates were calculated using a random-effects model. Between-study heterogeneity was assessed using the I^2^ statistic, and publication bias was evaluated using Egger’s test. *Results*: A total of 117 studies comprising 193,672 subjects were included. The pooled global prevalence of PD was 11.1% (95% CI: 8.0–14.2%) with substantial heterogeneity (I^2^ = 99.96%). PD prevalence was higher among individuals with pancreatic disease (18.7%) compared with cadaveric/autopsy studies (8.8%), healthy individuals (5.6%), and consecutive patients (4.7%). Both complete and incomplete PD were more common in subjects with pancreatic diseases. Among PD subtypes, type I was the most prevalent. Egger’s test demonstrated significant publication bias (*p* < 0.01). *Conclusions*: PD affects approximately one in ten individuals worldwide and appears more prevalent in patients with pancreatic diseases. However, this finding should be interpreted with caution due to potential selection bias from predominantly ERCP-based studies.

## 1. Introduction

Pancreas divisum (PD) is a common congenital malformation in the pancreatic duct system that occurs due to the absence of fusion between the ventral and dorsal ducts during development. Its prevalence ranges from 0.2% to 47% across studies [1,2]. The PD is categorized into two forms: complete and incomplete. Complete PD is characterized by a fully separate pancreatic ductal system within an undivided gland. It has been thought that complete PD arises from the inability of the ventral and dorsal pancreatic ducts to merge. Incomplete PD is a pancreatic abnormality characterized by insufficient communication between the ventral and dorsal pancreatic ducts.

The contribution of PD in the development of pancreatic diseases is controversial [3]. PD, although not considered a disease, is believed to be associated with certain pancreatic conditions or diseases in some cases [4,5]. One proposed theory suggests that a dorsal pancreatic duct, which is both larger and longer, opening through a relatively smaller or narrowed minor papilla, might not effectively drain pancreatic secretions, causing blockages in the flow [6]. This could result in elevated intraductal pressure and expansion of the dorsal duct, which, in turn, may give rise to abdominal discomfort and possibly pancreatitis. Additionally, it has been postulated that PD reduces the threshold for pancreatic disease from other risk factors of pancreatitis, including alcohol consumption and medications [7]. However, most individuals with PD do not exhibit symptoms related to their pancreas, leading to significant discussions about whether it is directly linked to pancreatitis or abdominal pain resembling pancreatic issues. Endoscopic retrograde cholangiopancreatography (ERCP) is the most commonly used method to diagnose PD. More recently, magnetic resonance cholangiopancreatography (MRCP) has been used with increasing frequency as a noninvasive alternative to ERCP [8]. It is worth noting that only patients with pain in the upper abdominal region or suspected of having pancreatic diseases are qualified for ERCP or MRCP. This data indicates that the increased prevalence of PD in pancreatic disease patients may be influenced by selection bias. In other words, the way patients are selected for the procedures may lead to a skewed understanding of the relationship between pancreatic diseases and PD.

Therefore, the aim of this study was to conduct a systematic review and meta-analysis to study the prevalence of PD in healthy individuals and those diagnosed with pancreatic diseases. The relationship between the presence of PD and the increased risk of pancreatic diseases was assessed using a meta-analysis.

## 2. Materials and Methods

### 2.1. Literature Search and Study Selection

**Figure 1:**
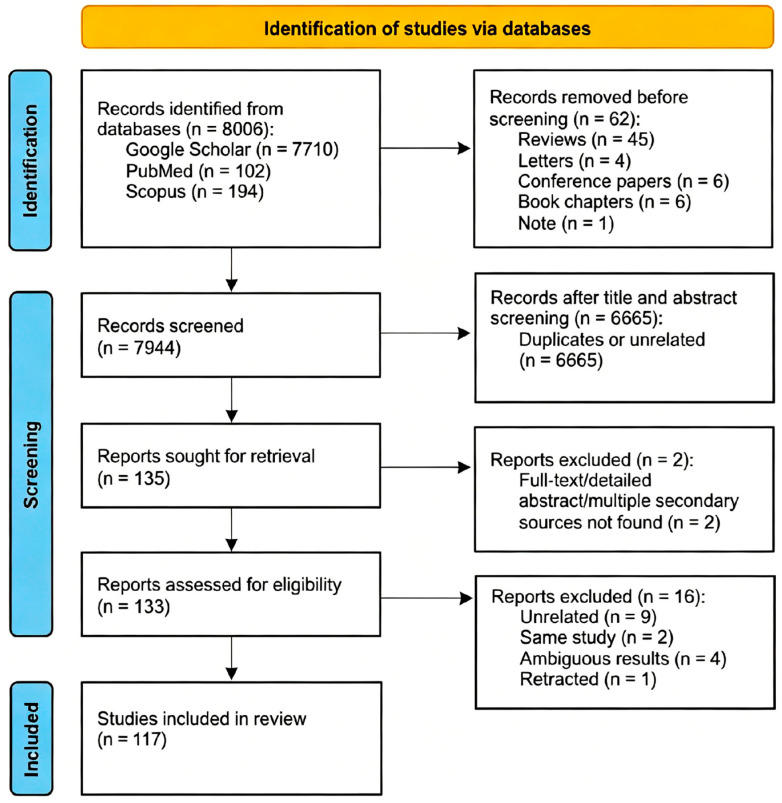
PRISMA flowchart summarizing the methodology of this systematic review.

The present study was registered and performed in accordance with the PRISMA 2020 statement [9]. This study was not registered on PROSPERO because it did not accept prevalence-based systematic reviews at the time that this work was conducted. A systematic literature search was conducted independently through three electronic databases, including Google, Scopus, and PubMed (Figure 1). Google Scholar was selected as a primary database due to its effectiveness in identifying studies within the grey literature, thereby enhancing the comprehensiveness of the search [10]. Combinations of the following keywords were used: pancreas divisum, prevalence, and incidence. Review articles, chapters, conference papers, and letters to the editor were excluded.

Screening was performed independently by two reviewers (W.S. and A.S.). Entries were subject to further review if all of the following information was reported: (1) the number of total subjects; (2) the number of subjects diagnosed with PD; and (3) the diagnostic method (ERCP, MRCP, cadaveric, and intraoperative). However, to ensure feasibility and relevance, only the first 100 pages of results from Google Scholar were screened. For other databases, all entries were screened. No restriction was imposed on language, study design, or date of publication. Studies were excluded if their full text or detailed abstract was not found. Studies were also excluded if their sample size was unclear. Automated translation was used for articles written in languages other than English. Two reviewers (W.S. and A.S.) independently evaluated all potential studies. Disagreement, if any, was resolved by discussion until a consensus was reached.

### 2.2. Risk of Bias Assessment and Meta-Analysis

The methodological quality of the included studies was assessed using the Anatomical Quality Assessment (AQUA) tool [11], which evaluates five key domains: study objectives and patient characteristics (domain 1), study design (domain 2), methodology description (domain 3), anatomical description (domain 4), and results reporting (domain 5). A domain was rated as “high risk” if more than two questions within that domain received a “yes” response. Two reviewers (L.Y. and A.S.) independently evaluated each study, and any discrepancies were resolved through discussion and consensus. The primary outcomes included the total number of subjects, the number of subjects diagnosed with PD, the diagnostic method, the history of pancreatic diseases, the demographic information of the subjects, and the geographical region. The secondary outcomes were overall PD prevalence, disease-based prevalence, and geographical region-based prevalence. A meta-analysis using DerSimonian–Laird (a random-effect model) was then applied. Between-study heterogeneity was evaluated using I^2^ statistics. A funnel plot of prevalence and standard error, along with Begg’s and Egger’s tests, was used to assess publication bias. Potential covariates influencing the secondary outcomes were evaluated by subgroup analysis. Differences within each subgroup were evaluated using Q-statistics. Pearson’s correlation coefficient (r) was used to assess the relationship between sample size and year of publication. Sensitivity analyses were performed by excluding studies classified as high risk of bias based on the AQUA tool, and pooled prevalence estimates were recalculated within key subgroups to assess the robustness of the findings. Stata version 19.5 (StataCorp, College Station, TX, USA) was used to perform meta-analysis and other statistical analyses. Statistical significance was established at *p* = 0.05. Raw data of the present study are available in Table S1.

## 3. Results

### 3.1. Overview of the Results of the Systematic Review

A total of 117 studies met the inclusion criteria (Table 1). No studies were excluded following the risk-of-bias assessment using the AQUA tool, although seven studies in total were rated as ‘high risk’ in domains 2, 3, and 4 (Table S1). The systematic review encompassed patients from diverse continents. The highest proportion of patients originated from North America, accounting for 35.6% of the total patient population. Asia followed with 29.7%, and Europe contributed 27.7%. This global distribution highlights a significant concentration of studies from North America and Asia, with moderate representation from Europe.

Various diagnostic methods were employed across the studies. The most frequently used technique was endoscopic retrograde cholangiopancreatography (ERCP), utilized in 72.0% of studies. Magnetic resonance cholangiopancreatography (MRCP) was the second most common method, featured in 10.3% of studies, while Cadaveric studies accounted for 3.7%. This indicates a predominant reliance on ERCP for the diagnosis and evaluation of pancreas divisum.

In terms of study design and populations, 60 studies (51.3%) were conducted in consecutive patients. Forty-three studies (36.7%) involved patients with suspected or known pancreatic diseases. Pancreatic diseases included various forms of pancreatitis, namely acute, chronic, chronic calcifying, recurrent, and idiopathic pancreatitis, as well as combined or unspecified categories, along with other conditions such as pancreatic cancer and pancreatic duct infection (Table S1). Five studies (4.3%) were conducted in cadavers or autopsied specimens.

### 3.2. Prevalence of Pancreas Divisum

**Figure 2:**
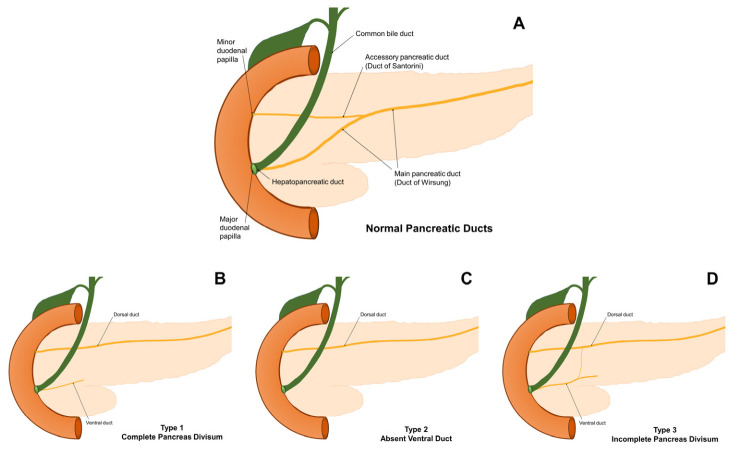
Anatomical illustration of different types of pancreas divisum. (**A**) Normal pancreatic ducts; (**B**) Complete pancreas divisum; (**C**) Absent ventral duct; (**D**) Incomplete pancreas divisum.

A total of 117 studies with 193,672 subjects yielded an overall prevalence of pancreas divisum (PD) of 11.1% (95% CI: 8.0–14.2%, I^2^ = 99.96%) (Figure 2). The test for heterogeneity was significant (Q = 49,838.32, *p* < 0.001), indicating substantial variability among studies. When stratified by study population, the highest prevalence of PD was reported in studies involving known or suspected pancreatic disease (18.7%, 95% CI: 11.5–25.9%, I^2^ = 99.72%), followed by cadaveric/autopsied pancreas studies (8.8%, 95% CI: 0.4–17.3%, I^2^ = 92.94%). Studies on consecutive patients reported a lower prevalence (4.7%, 95% CI: 3.7–5.6%, I^2^ = 99.58%), while studies focusing on individuals with no history of pancreatitis showed a prevalence of 5.6% (95% CI: 0–15.4%, I^2^ = 96.22%). The difference in prevalence across the aforementioned subgroups was statistically significant (Q = 15.17, *p* = 0.002). Sensitivity analyses (Table S1) revealed no meaningful differences in pooled prevalence estimates after excluding studies with a higher risk of bias, indicating that the findings are robust and not driven by methodologically weaker studies, and that the overall conclusions remain stable under different analytical assumptions.

In terms of geographical population, the highest prevalence was observed in North America (14.5%, 95% CI: 8.6–20.4%, I^2^ = 99.95%), followed by Europe (10.2%, 95% CI: 3.7–16.7%, I^2^ = 99.82%), and Oceania (8.2%, 95% CI: 1.0–15.5%, I^2^ = 75.44%). South America and Asia reported lower prevalence rates at 6.5% (95% CI: −1.7–14.7%, I^2^ = 70.48%) and 7.1% (95% CI: 3.1–11.2%, I^2^ = 99.97%), respectively. The highest prevalence was reported in studies utilizing intraoperative diagnosis (55.7%, 95% CI: 0–100.0% I^2^ = 99.00%), followed by combined ERCP or MRCP studies (15.5%, 95% CI: 10.9–20.0% I^2^ = 88.57%) and ERCP alone (7.9%, 95% CI: 5.0–10.8%). Studies using MRCP alone reported a prevalence of 8.5% (95% CI: 5.6–11.3% I^2^ = 99.97%), while cadaveric studies found a prevalence of 10.2% (95% CI: 0–21.9% I^2^ = 92.61%). High heterogeneity was observed across studies (I^2^ = 99.96%, τ^2^ = 0.028), suggesting substantial variability in prevalence estimates. Leave-one-out meta-analysis did not identify any influential study or potential outlier.

**Figure 3:**
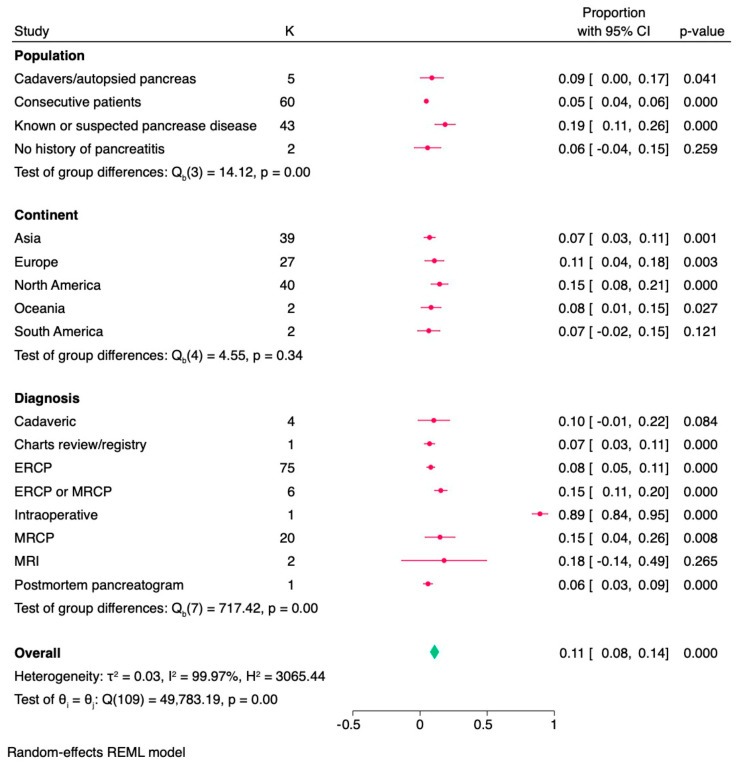
Subgroup forest plot showing the prevalence of pancreas divisum across population types, continents, and diagnostic tools used.

Type-based PD prevalence is presented in Table 2. Twenty-two studies classified the PD into complete and incomplete subtypes (Figure 2). Complete PD was present in 7.9% (95% CI: 2.8–13.0%, I^2^ = 96.19%) of subjects with pancreatic diseases, and incomplete PD was found in 9.7% (95% CI: 0–21.9%, I^2^ = 99.98%). In healthy and consecutive subjects, the prevalence was lower: 1.6% (95% CI: 0.5–2.7%, I^2^ = 99.43%) for complete PD and 0.7% (95% CI: 0.4–0.9%, I^2^ = 93.50%) for incomplete PD. Subgroup meta-analysis is presented in Figure 3. Subtype analysis in healthy subjects showed type I as the most common (2.5%, 95% CI: 0.5–4.4%, I^2^ = 99.40%), followed by type II (0.4%, 95% CI: 0.0–0.9%, I^2^ = 98.01%) and type III (0.4%, 95% CI: 0.2–0.7%, I^2^ = 83.63%). Among those with pancreatic diseases, type I remained the most prevalent (17.6%, 95% CI: 0–47.0%, I^2^ = 99.97%), followed by type II (5.5%, 95% CI: 0–14.1%, I^2^ = 99.81%) and type III (0.9%, 95% CI: 0.5–1.2%, I^2^ = 23.90%).

### 3.3. Publication Bias

**Figure 4:**
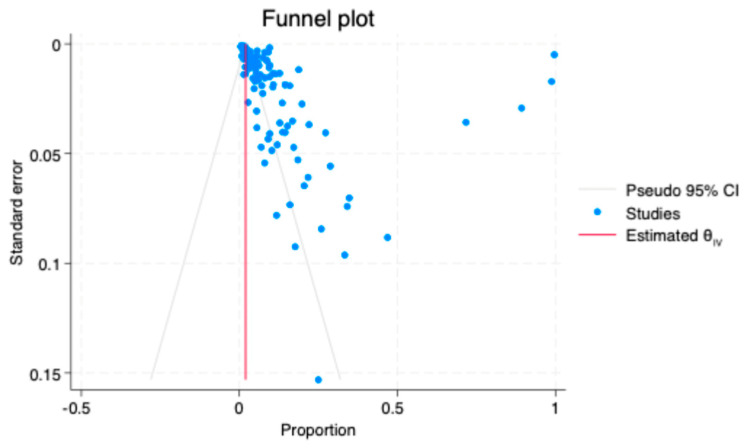
Funnel plot for publication bias.

A funnel plot was generated to assess potential publication bias among the included studies (Figure 4). The plot shows an asymmetrical distribution of studies around the estimated effect size, with a noticeable clustering of studies on the left side of the plot and outside the pseudo 95% confidence limits. This asymmetry suggests the presence of publication bias. Egger’s regression test confirmed the presence of small-study effects, with a significant intercept (β_1_ = 2.7, SE = 0.64, z = 4.21, *p* < 0.01), indicating substantial publication bias. No significant correlation was found between sample size and year of publication (r = 0.053, *p* = 0.567).

## 4. Discussion

This study systematically located previous research on the prevalence of PD, calculated its overall prevalence, and identified factors associated with its occurrence. Although a recent systematic review [111] examined the frequency and morphometry of pancreatic ductal system variations, it did not include the prevalence of PD and its subtypes as part of its objectives. Our results indicated that PD was present in 10.2% of the general population. However, significant between-study heterogeneity was observed, indicating the need for further investigation to identify factors influencing PD occurrence. Therefore, subgroup analysis was carried out to examine whether factors, including continent and diagnostic method, could influence PD prevalence. We found that PD was most prevalent in North American populations (14.6%) and was least prevalent in Asian populations (2.5%), which suggests that race or underlying genetic differences may be a contributing factor to PD. The funnel plot of PD prevalence against sample size suggested that the results of this meta-analysis were likely influenced by the small-study effect or publication bias, which may also explain the considerable heterogeneity among studies. The small-study effect describes the tendency of smaller studies to report higher effect sizes (prevalence) than larger studies (Sterne et al. [112]), leading to a distortion in the overall prevalence estimate. Yurasakpong et al. [113] were the first to identify this phenomenon in anatomical research. No significant correlation was observed between sample size and year of publication (r = 0.053, *p* = 0.567), suggesting that this small-study effect is unlikely to be explained by temporal trends in study design or increasing sample sizes over time. To avoid this effect, we recommend that future anatomical studies use an appropriate sample size to ensure an accurate prevalence estimate. Given the extremely high heterogeneity observed (I^2^ = 99.96%), the pooled prevalence should be interpreted with caution, as it reflects an average across highly diverse study populations and methodologies rather than a precise estimate. The appropriateness of pooling under such conditions is therefore limited, and the results are better understood as indicative of overall trends. Potential sources of heterogeneity likely include differences in diagnostic modalities (e.g., ERCP, MRCP, and cadaveric studies), variation in population selection (e.g., healthy, consecutive, or disease-specific cohorts), and temporal changes in diagnostic practices. Although subgroup analyses were performed to partially account for these factors, substantial residual heterogeneity remained, highlighting the need for more standardized study designs and diagnostic criteria in future research.

We found that PD was 3.3 times more prevalent in individuals with known or suspected pancreatic diseases, suggesting a contributing role in the development of pancreatic diseases. It has been proposed that when the dominant dorsal pancreatic duct is relatively enlarged and drains through a narrower or stenotic minor papilla, the resulting anatomical configuration may impair the outflow of pancreatic secretions, causing functional obstruction. This can lead to increased intraductal pressure and distention of the dorsal duct, which may contribute to abdominal pain and, in some cases, pancreatitis—a phenomenon referred to in the literature as “dominant duct syndrome” [32,114]. Manometric studies in patients with PD have revealed elevated pressures within the dorsal duct and minor papilla compared to the ventral duct and major papilla, suggesting localized ductal hypertension [7]. It has further been hypothesized that such obstruction may be intermittent, potentially caused by episodic blockage of the minor papilla by proteinaceous material in pancreatic secretions [115]. Nonetheless, most individuals with a dilated dorsal duct in the context of PD remain asymptomatic, indicating a weak association between ductal obstruction and clinical symptoms [115]. Importantly, epidemiological data indicate that over 95% of individuals with PD are asymptomatic, and the anomaly is often detected incidentally [116], with only a small proportion developing pancreatitis [117]. Therefore, the increased prevalence of PD observed in diseased cohorts should be interpreted cautiously, as it may partly reflect selection bias arising from the investigation of symptomatic patients rather than a direct causal relationship, with PD more likely acting as a predisposing or cofactor condition in the presence of additional risk factors.

The use of ERCP with minor papillotomy has traditionally been considered a therapeutic option for patients with PD presenting with recurrent acute pancreatitis, based largely on observational studies suggesting improved ductal drainage and reduced recurrence rates. In particular, retrospective studies have reported substantial reductions in pancreatitis episodes following endoscopic minor papilla sphincterotomy in selected patients, especially those with anatomical variants such as Santorini’s cyst [118]. However, more recent high-quality evidence has challenged this paradigm. A multicenter randomized controlled trial demonstrated that ERCP with minor papillotomy did not significantly reduce the risk of recurrent pancreatitis compared with sham intervention, while also being associated with a notable risk of post-procedural pancreatitis [119]. These findings highlight the uncertainty regarding the clinical benefit of endoscopic intervention in PD.

The present study has a few limitations. The included studies exhibited high heterogeneity even after subgroup analyses. Potential contributing factors include the small-study effect, inter-observer variability, or random variation. Determining the sex-based prevalence of PD was not feasible due to the limited number of studies reporting the sex of their subjects. While Google Scholar provides broad coverage, its search results lack repeatability [120]. The inclusion of both confirmed and suspected pancreatic disease cases may have introduced misclassification bias, as individuals with suspected pancreatic disease may not have a definitive diagnosis, potentially affecting the accuracy of the reported prevalence estimates. The absence of studies in general pediatric populations limited meaningful age-based analysis, as available data were restricted to children with suspected or known pancreatic diseases, potentially confounding the results by underlying disease status. Further studies using well-defined, population-based cohorts and longitudinal designs are needed to clarify whether pancreas divisum represents a causal factor or an incidental anatomical variant in pancreatic disease.

## 5. Conclusions

PD is a relatively common congenital anomaly, affecting approximately one in ten individuals globally. Its prevalence appears higher in patients with pancreatic disorders; however, this observation should be interpreted with caution, as it may be influenced by selection bias arising from predominantly ERCP-based studies that oversample symptomatic populations. Therefore, while PD may have clinical relevance in selected cases, its role as a causal factor in pancreatic disease remains uncertain, and further well-designed population-based and prospective studies are required to clarify its true significance.

## Figures and Tables

**Table 1:** Demographic background of studies included in this review.

Study	Population Category	Age	Country	Method
Gregg [12]	Consecutive patients	Adults	USA	ERCP
Cotton [13]	Consecutive patients	Adults	UK	ERCP
Tulassay et al. [14]	Consecutive patients	Adults	Hungary	ERCP
Richter [15]	Consecutive patients	Adults	USA	ERCP
Sahel et al. [16]	Consecutive patients	Adults	France	ERCP
Sahel et al. [16]	Known or suspected pancreatic disease	Adults	France	ERCP
Sahel et al. [16]	Known or suspected pancreatic disease	Adults	France	ERCP
Cooperman et al. [17]	Consecutive patients	Adults	USA	ERCP
Britt et al. [18]	Consecutive patients	Adults	USA	ERCP
Warshaw et al. [19]	Known or suspected pancreatic disease	Adults	USA	ERCP
Forbes et al. [20]	Known or suspected pancreatic disease	Children	UK	ERCP
Shibuya et al. [21]	Consecutive patients	Adults	Japan	ERCP
Delhaye et al. [22]	Consecutive patients	Adults	Belgium	ERCP
[23]	Consecutive patients	Adults	Japan	ERCP
Sugawa et al. [24]	Consecutive patients	Adults	USA	ERCP
Agha et al. [25]	Known or suspected pancreatic disease	Adults	USA	ERCP
Agha et al. [25]	Known or suspected pancreatic disease	Adults	USA	ERCP
Allendorph et al. [26]	Consecutive patients	Adults	USA	ERCP
Igarashi et al. [27]	Consecutive patients	Adults	Japan	ERCP
Hayakawa et al. [28]	Consecutive patients	Adults	Japan	ERP
Bernard et al. [29]	Consecutive patients	Adults	France	ERCP
Brenner et al. [30]	Consecutive patients	Adults	Australia	ERCP
Brenner et al. [30]	Known or suspected pancreatic disease	Adults	Australia	ERCP
Lindström et al. [31]	Consecutive patients	Adults	Sweden	ERCP
Warshaw et al. [32]	Known or suspected pancreatic disease	Adults	USA	Intraoperative
Saowaros [33]	Consecutive patients	Adults	Thailand	ERCP
Ladas et al. [34]	Consecutive patients	Adults	Greece	ERCP
Brown et al. [35]	Consecutive patients	Adults	USA	ERCP
Lemmel et al. [36]	Known or suspected pancreatic disease	Children	-	ERCP
Brown et al. [35]	Known or suspected pancreatic disease	Children	USA	ERCP
Guelrud et al. [37]	Known or suspected pancreatic disease	Children	Venezuela	ERCP
Suga et al. [38]	Consecutive patients	Adults	Japan	ERCP
Narisawa et al. [39]	Consecutive patients	Adults	Japan	ERCP
[40]	Consecutive patients	Adults	Italy	ERCP
Barthet et al. [41]	Known or suspected pancreatic disease	Adults	France	ERCP
Dhar et al. [42]	Consecutive patients	Adults	India	ERCP
Guelrud [43]	Consecutive patients	Children	Venezuela	ERCP
Bret et al. [44]	Known or suspected pancreatic disease	Adults	Canada	MRCP
Stimec et al. [45]	Consecutive patients	Adults	Yugoslavia	ERCP
Stimec et al. [45]	Cadavers/autopsied pancreas	Adults	Yugoslavia	Postmortem pancreatogram
Schimanski et al. [46]	Known or suspected pancreatic disease	Adults	Germany	ERCP
Lee et al. [47]	Consecutive patients	Adults	Korea	ERCP
Ueno et al. [48]	Known or suspected pancreatic disease	Adults	Japan	MRCP
Morgan et al. [49]	Consecutive patients	Adults	USA	ERCP
Jacob et al. [50]	Consecutive patients	Adults	USA	ERCP
Khandekar et al. [51]	Consecutive patients	Adults	USA	ERCP
Manfredi et al. [52]	Known or suspected pancreatic disease	Adults	Italy	S-MRCP
Neblett III et al. [53]	Known or suspected pancreatic disease	Children	USA	ERCP
Eisendrath et al. [54]	Known or suspected pancreatic disease	Adults	Belgium	ERCP
Boerma et al. [55]	Known or suspected pancreatic disease	Adults	Netherlands	ERCP
Kim et al. [56]	Consecutive patients	Adults	Korea	ERCP
Matos et al. [57]	No history of pancreatitis	Adults	Belgium	ERCP
Zoepf et al. [58]	-	Adults	Germany	ERCP
Zoepf et al. [58]	-	Adults	Germany	ERCP
Kim et al. [1]	Consecutive patients	Adults	Korea	ERCP
Mortelé [59]	Consecutive patients	Adults	USA	MRCP
Mortelé [59]	Consecutive patients	Adults	USA	MRCP
Lai et al. [60]	Consecutive patients	Adults	USA	ERCP
Kamisawa [61]	Consecutive patients	Adults	Japan	ERCP
Cheng et al. [62]	Consecutive patients	Children	USA	ERCP
Soto et al. [63]	Consecutive patients	Adults	USA	ERCP
Kin et al. [64]	Cadavers/autopsied pancreas	Adults	USA	Cadaveric
Fischer et al. [65]	Known or suspected pancreatic disease	Adults	USA	ERCP
Tessier et al. [66]	Known or suspected pancreatic disease	Adults	-	ERCP
Ewald et al. [67]	Known or suspected pancreatic disease	-	Germany	Charts review/registry
Kamisawa et al. [68]	Consecutive patients	Adults	Japan	ERCP
Nishino et al. [69]	Consecutive patients	Adults	Japan	ERCP
Alempijevic et al. [70]	Cadavers/autopsied pancreas	Adults	Serbia	Cadaveric
Vaughan et al. [71]	Consecutive patients	Adults	USA	ERCP
Fogel et al. [2]	-	Adults	-	ERCP
Bang et al. [72]	Consecutive patients	Adults	Korea	ERCP
Kamisawa et al. [73]	Consecutive patients	Adults	Japan	ERCP
Alazmi et al. [74]	Known or suspected pancreatic disease	Adults	USA	ERCP
Sánchez-Ramírez et al. [75]	Known or suspected pancreatic disease	Children	Mexico	ERCP
Chalazonitis et al. [76]	Known or suspected pancreatic disease	Children	USA	ECRP or MRCP
De Filippo et al. [77]	Known or suspected pancreatic disease	Adults	Italy	MRCP
Carnes et al. [78]	Consecutive patients	Adults	USA	ERCP
Terui et al. [79]	Known or suspected pancreatic disease	Children	Japan	ERCP
Gonoi et al. [80]	Consecutive patients	Adults	Japan	MRI
Gonoi et al. [80]	Known or suspected pancreatic disease	Adults	Japan	MRI
Moffatt et al. [81]	Consecutive patients	Adults	USA	ERCP
Mosler et al. [82]	Consecutive patients	Adults	USA	S-MRCP
Asayama et al. [83]	Known or suspected pancreatic disease	Adults	Japan	ERCP
Sultan et al. [84]		Children	USA	Charts review/registry
Bertin et al. [85]	Known or suspected pancreatic disease	Adults	France	MRCP
Wang et al. [8]	Consecutive patients	Adults	China	ERCP or MRCP
Rustagi et al. [86]	Consecutive patients	Adults	USA	ERCP
Kushnir et al. [87]	Consecutive patients	Adults	USA	MRCP
White et al. [88]	Cadavers/autopsied pancreas	-	USA	Cadaveric
Bülow et al. [89]	Consecutive patients	Adults	Germany	MRCP
Boninsegna et al. [90]	Known or suspected pancreatic disease	Adults	Italy	S-MRCP
Ballard et al. [91]	Known or suspected pancreatic disease	Adults	USA	ERCP or MRCP
Gupta et al. [92]	Known or suspected pancreatic disease	Adults	India	MRCP
Gupta et al. [92]	No history of pancreatitis	Adults	India	MRCP
Taj et al. [93]	Consecutive patients	Adults	Pakistan	ERCP
Angsuwatcharakon et al. [94]	Cadavers/autopsied pancreas	Adults	Thailand	Cadaveric
Lu et al. [95]	Known or suspected pancreatic disease	Children	China	ERCP
Adibelli et al. [96]	Consecutive patients	Adults	Turkey	ERCP
Adibelli et al. [97]	Known or suspected pancreatic disease	Adults	Turkey	MRCP
Moran et al. [98]		Adults	USA	Intraoperative
Moran et al. [98]	Consecutive patients	Adults	USA	ERCP
Lin et al. [3]	Pancreatitis	Children	USA	ERCP or MRCP
Deng et al. [99]	Consecutive patients	Children	China	ERCP
Kawaguchi et al. [100]	Consecutive patients	Adults	Japan	ERCP
Baş et al. [101]	Known or suspected pancreatic disease	Adults	Turkey	ERCP or MRCP
Qin et al. [102]	Known or suspected pancreatic disease	Children	China	ERCP
Zhang et al. [103]	Known or suspected pancreatic disease	Children	China	ERCP and EDCT
Saad et al. [104]	Known or suspected pancreatic disease	Children	USA	MRCP
Heinzman et al. [105]	Known or suspected pancreatic disease	Children	USA	ERCP or MRCP
Gaitanidis et al. [106]	Consecutive patients	Adults	USA	MRCP
Mao et al. [107]	Consecutive patients	Adults	China	MRCP
Choudhury et al. [108]	Known or suspected pancreatic disease	Adults	India	MRCP
Kotha et al. [109]	Known or suspected pancreatic disease	Adults	India	MRCP
Pavan et al. [110]	Known or suspected pancreatic disease	Children	India	MRCP

**Table 2:** Prevalence estimates of subtypes of pancreas divisum in consecutive and healthy subjects versus subjects with pancreatic diseases.

Types	Consecutive and Healthy Subjects			Subjects with Pancreatic Diseases		
	n	Prevalence	I ^2^	n	Prevalence	I ^2^
Complete	13	1.6% (0.5–2.7%)	99.43%	7	7.9% (2.8–13.0%)	96.19%
Incomplete	9	0.7% (0.4–0.9%)	93.50%	6	9.7% (0–21.9%)	99.98%
Type I	5	2.5% (0.5–4.4%)	99.40%	3	17.6% (0–47.0%)	99.97%
Type II	4	0.4% (0.0–0.9%)	98.01%	3	5.5% (0–14.1%)	99.81%
Type III	5	0.4% (0.2–0.7%)	83.63%	3	0.9% (0.5–1.2%)	23.90%

## Data Availability

The original contributions presented in this study are included in the article/Supplementary Material. Further inquiries can be directed to the corresponding author.

## Supplementary Materials

The following supporting information can be downloaded at: https://www.mdpi.com/article/10.3390/medicina62050953/s1, Table S1: Raw data for meta-analysis.

## Abbreviations

The following abbreviations are used in this manuscript:

PD	Pancreas divisum
ERCP	Endoscopic retrograde cholangiopancreatography
MRCP	Magnetic resonance cholangiopancreatography
CI	Confidence interval
